# Stroke Experiences in Weblogs: A Feasibility Study of Sex Differences

**DOI:** 10.2196/jmir.2838

**Published:** 2014-03-19

**Authors:** Sukjin Koh, Andrew S Gordon, Christopher Wienberg, Sara O Sood, Stephanie Morley, Deborah M Burke

**Affiliations:** ^1^Department of Lingusitics and Cognitive SciencePomona CollegeClaremont, CAUnited States; ^2^Institute for Creative TechnologiesUniversity of Southern CaliforniaLos Angeles, CAUnited States; ^3^Department of Computer SciencePomona CollegeClaremont, CAUnited States; ^4^Department of PsychologyClaremont McKenna CollegeClaremont, CAUnited States

**Keywords:** cerebral stroke, signs and symptoms, sex differences, Internet, blogging

## Abstract

**Background:**

Research on cerebral stroke symptoms using hospital records has reported that women experience more nontraditional symptoms of stroke (eg, mental status change, pain) than men do. This is an important issue because nontraditional symptoms may delay the decision to get medical assistance and increase the difficulty of correct diagnosis. In the present study, we investigate sex differences in the stroke experience as described in stories on weblogs.

**Objective:**

The goal of this study was to investigate the feasibility of using the Internet as a source of data for basic research on stroke experiences.

**Methods:**

Stroke experiences described in blogs were identified by using StoryUpgrade, a program that searches blog posts using a fictional prototype story. In this study, the prototype story was a description of a stroke experience. Retrieved stories coded by the researchers as relevant were used to update the search query and retrieve more stories using relevance feedback. Stories were coded for first- or third-person narrator, traditional and nontraditional patient symptoms, type of stroke, patient sex and age, delay before seeking medical assistance, and delay at hospital and in treatment.

**Results:**

There were 191 relevant stroke stories of which 174 stories reported symptoms (52.3% female and 47.7% male patients). There were no sex differences for each traditional or nontraditional stroke symptom by chi-square analysis (all *P*s>.05). Type of narrator, however, affected report of traditional and nontraditional symptoms. Female first-person narrators (ie, the patient) were more likely to report mental status change (56.3%, 27/48) than male first-person narrators (36.4%, 16/44), a marginally significant effect by logistic regression (*P*=.056), whereas reports of third-person narrators did not differ for women (27.9%, 12/43) and men (28.2%, 11/39) patients. There were more reports of at least 1 nontraditional symptom in the 92 first-person reports (44.6%, 41/92) than in the 82 third-person reports (25.6%, 21/82, *P*=.006). Ischemic or hemorrhagic stroke was reported in 67 and 29 stories, respectively. Nontraditional symptoms varied with stroke type with 1 or more nontraditional symptoms reported for 79.3% (23/29) of hemorrhagic stroke patients and 53.7% (36/67) of ischemic stroke patients (*P*=.001).

**Conclusions:**

The results replicate previous findings based on hospital interview data supporting the reliability of findings from weblogs. New findings include the effect of first- versus third-person narrator on sex differences in the report of nontraditional symptoms. This result suggests that narrator is an important variable to be examined in future studies. A fragmentary data problem limits some conclusions because important information, such as age, was not consistently reported. Age trends strengthen the feasibility of using the Internet for stroke research because older adults have significantly increased their Internet use in recent years.

## Introduction

### Background

The Internet has become a valuable tool in clinical medicine, both as a source of health information and by providing online interventions designed to improve health [1]. Moreover, the Internet also offers a research opportunity through the wealth of information available from search queries or social network posts. For example, the type and frequency of search queries concerning infectious disease have been associated with outbreaks of these diseases [2,3] and analysis of weblog stories has revealed young adults’ mental health concerns [4]. Following this infodemiology approach [5], the present research investigates the feasibility of analyzing weblog posts about cerebral stroke experiences as a source of evidence to test a hypothesis in basic medical research: whether or not there are sex differences in cerebral stroke symptoms.

Our interest in variation between men and women in stroke experiences derives from reports based on hospital records of a variety of sex differences ranging from experience of symptoms to treatment, and from the possibility that these differences may influence stroke outcome. There is evidence that women experience delays from the onset of symptoms to diagnosis and treatment of stroke compared to men [6,7], although this difference is not consistently reported [8]. Such delays are a concern because they may obviate certain treatments that require rapid administration after onset of symptoms. Indeed, women are less likely than men to receive thrombolytic treatment, which may be because women are more likely to experience delay after the onset of symptoms that is beyond the 3-hour treatment window [6,9]. Women receive fewer diagnostic tests than men do, even when controlling for age, ethnicity, insurance status, and risk factors [10]. Moreover, outcomes of stroke are poorer for women compared to men [7,11-14]. Women’s older age at the time of stroke is a likely factor, but delays in treatment and the type of treatment may also be a factor as well [15].

In sum, there are several factors that may contribute to sex differences in type and delay of treatment. We focus on one of them: There are reports that women experience more nontraditional symptoms of stroke than men and this may make correct diagnosis more difficult for women [9,15,16].

### Sex Differences in Stroke Symptoms

Stroke is the fourth leading cause of death in the United States, [17] with more women than men dying of stroke each year [14,17]. Traditional symptoms of stroke include hemibody numbness and paresis, language disorders in comprehension and/or production, dysarthria, diplopia and other visual disturbances, facial weakness, ataxia, vertigo, and imbalance. Nontraditional symptoms of stroke include pain, mental status change (disorientation, confusion, or loss of consciousness), headache, lightheadedness, other general neurological symptoms (nausea, hiccups, and nonfocal weakness), and some nonneurological symptoms (chest pain, palpitations, and shortness of breath) [15,18,19]. Although headache has been reported as a symptom of ischemic and hemorrhagic strokes [20,21], headache has been considered a nontraditional symptom because it is not a focal neurological sign and it is common in a number of health problems [18].

Mental status change is the nontraditional stroke symptom most consistently reported as occurring more frequently in women than men [11,13,15,18,22,23]. Other nontraditional symptoms that have been reported as more frequent in women than men, although with less consistency, are pain other than headache [15]; (see [8] for a marginally significant difference), difficulty swallowing [11,24], headache [20], visual disturbances [24], and generalized weakness [23]. In contrast, women were significantly less likely than men to report traditional symptoms of dizziness or problems with walking or balance [15,16,23,25]. Indeed, in one study, women were less likely than men to experience any traditional stroke symptoms or to suspect stroke [8].

### The Present Study

In this study, we investigate the feasibility of using stroke experiences described in weblogs as a source of data on sex differences in traditional and nontraditional symptoms. We used newly developed software that identified personal stories on the blogosphere that were relevant to a prototype story. We then analyzed symptoms described in the blogs by a stroke survivor or a third-person narrator. This follows hospital studies of stroke patients that included symptom reports by patients or third parties [16,18,23]. We predict that the identity of the narrator will be significant in reports of mental status change, a symptom often reported as more common in women patients. Mental change may have no effect observable to a third party unlike traditional symptoms, such as hemibody weakness, speech impairment, or loss of balance. Moreover, a patient’s communication of internal mental states to a third party may be constrained by speech impairments associated with stroke. Thus, we will test for the first time whether or not changes in mental status are more likely to be reported as a symptom by a first-person than a third-person narrator, and whether there are sex differences in these reports.

We anticipated that descriptions of stroke experiences would vary widely in the information supplied because of the absence of a prescribed format for blogs, but that salient aspects of the experience, such as symptoms, the identity of the narrator, and delay in treatment, would be included in most descriptions. We also anticipated that the patient population would over- and underrepresent certain demographic groups in the population of stroke patients inasmuch as younger adults use blogs more than older adults and women bloggers make up the majority of this younger age group, whereas men bloggers make up the majority in the older age group [26]. These constraints will be considered in evaluating the feasibility of analysis of weblog data in testing hypotheses concerning sex differences in stroke symptoms.

## Methods

### Sampling Blogs

**Figure 1:**
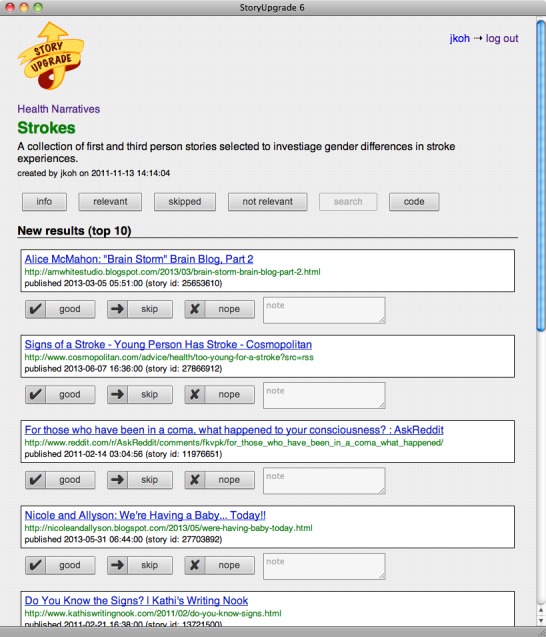
Screenshot of StoryUpgrade showing search results for stroke narratives.

Stroke experiences described in weblogs were identified using a program called StoryUpgrade [27]. StoryUpgrade is a system we developed to aid in the retrieval of stories that describe classes of activities or events that we wish to analyze (eg, stories about protest rallies, car crashes, or stroke experiences). The system is supplied with a constant stream of stories pulled from the Web. Each day, the system downloads 1.5 million English-language weblog posts. The system then applies supervised machine learning techniques to classify these posts as personal stories. This classifier has a precision of 0.66 and recall of 0.5, meaning that it detects approximately half of all English-language personal stories, but one-third of posts it considers stories are actually not [28]. These posts are then indexed by using an off-the-shelf information retrieval platform, enabling them to be searched quickly. Figure 1 shows an example set of search results from the StoryUpgrade system. For the present study, the system was backed by 17.4 million personal stories posted to weblogs in 2010 and 2011.

In addition to enabling search queries, the StoryUpgrade system allows a user to provide feedback about the relevance of the retrieved stories to the user’s information need. A user can mark a story as relevant, irrelevant, or the user can simply skip the story. This feedback is then incorporated into the search system, allowing it to retrieve increasingly relevant stories. To accomplish this, the StoryUpgrade system uses the Rocchio algorithm [29]. This technique incorporates the user’s relevance feedback, encoding the information about story relevance provided by the user directly into the query. Words from stories the user considers relevant are weighed heavily, and words from stories the user considers irrelevant are weighed less heavily. This process modifies the original query to be more similar to relevant stories and less similar to irrelevant stories. The skip category allows a user to remove a story from the queue of stories to be judged without it impacting the weights of words in the query [30].

For this study, we wrote a fictional prototype story to be used as a query to the StoryUpgrade system. This prototype story was written in a colloquial style typical of blogs. It described a stroke experience including keywords for traditional and nontraditional symptoms (eg, “my speech did not make sense and was slurred,” “could not pick up my arm or leg on one side,” “I felt confused”), as well as “911,” “emergency room,” “paramedics,” and “diagnosed with stroke.” These keywords were selected based on symptoms and events that appear in hospital admission interviews or medical records of stroke patients [8,15,23]. The prototype story is presented in Multimedia Appendix 1.

The technique of writing a prototype story is one that has worked well with the StoryUpgrade system in other contexts. It is effective because it seeds the system with a vocabulary similar to what an actual blogger might use when writing a relevant story. An important concern of this technique is how it may bias the search results toward stories that feature details and phrasing included in the prototype story. Although this bias must be taken under consideration when analyzing the stories retrieved by StoryUpgrade, the bias is mitigated by the relevance feedback system previously described. As a StoryUpgrade user marks stories as relevant, the vocabulary from these stories is incorporated into the search query, casting a wider net for relevant stories. For instance, an initially retrieved story may describe the author experiencing slurred speech, a symptom described in the prototype story. This same story may describe vertigo, a symptom not included in the prototype story, or the author may say she “couldn’t think clearly,” describing confusion in a way that was not done in the prototype story. Although the initially retrieved stories may be biased toward the initial query, this bias is reduced as the user marks stories as relevant.

In this study, all stories retrieved by StoryUpgrade were read by 2 of the authors and marked as relevant if they contained information about a stroke experience in either a first- or third-person account, and marked as irrelevant if the story was not about a stroke. Irrelevant stories were dropped from further analysis. Stories that did not identify the sex of the stroke patient or were in blogs that denied access, such as expired pages or blogs requiring a subscription or permission, were ignored by being placed in the skip category (281 stories in this study). Using this procedure, we identified 191 relevant stories and 244 irrelevant stories.

### Coding

We developed codes for first- or third-person narrator, whether or not a third-person narrator witnessed the event, the relation of the third-person narrator to the patient, patient sex and age, patient symptoms, assistance to obtain medical attention (eg, 911) , delay before seeking assistance, delay at hospital, treatment, and stroke outcome. If the specific age of the patient was not available, age range was coded if explicit information was available.

**Figure 2:**
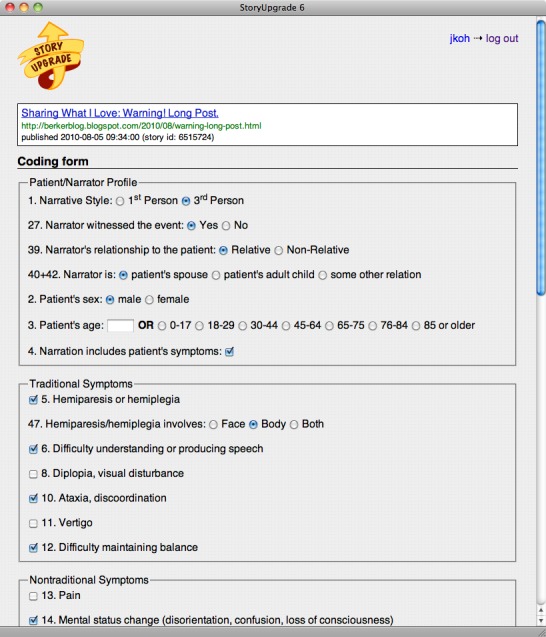
Screenshot of StoryUpgrade annotation tool for coding stroke narratives.

Classification of symptoms as traditional or nontraditional was based on the American Stroke Association’s stroke warning signs [31] as well as symptom classifications used in previous research on sex differences [15,18,19]. Traditional symptoms were hemiparesis/hemiplegia (for body, for face, or both body and face), impaired speech or comprehension, visual disturbance, ataxia/discoordination, vertigo, and difficulty with balance. Nontraditional symptoms were pain (excluding headache), mental status change (disorientation, confusion, loss of consciousness), headache, lightheadedness, other neurologic symptoms (nausea, hiccups, nonfocal weakness), and nonneurologic symptoms (chest pain, palpitations, shortness of breath). Although the American Stroke Association identifies confusion as a symptom, we categorized it as nontraditional consistent with previous research on sex differences. Each traditional and nontraditional symptom was coded for each patient as a dichotomous variable (reported or not reported). Symptoms were coded that occurred in the interval from the onset of the stroke experience until medical assistance was secured. Two of the authors coded all stories. An example of the form used to code each story is shown in Figure 2. Initial agreement in codes across the 2 coders was 83% and discrepancies in codes were discussed and resolved.

### Statistical Analysis

Each relevant story was assigned to a category for patient sex, stroke type, and type of narrator, with third-person narrators also categorized by whether or not they witnessed the stroke and their relation to the patient. The frequency of occurrence of each traditional and nontraditional symptom was compared between men and women and between ischemic and hemorrhagic stroke types using chi-square tests. Logistic regression was used to compare nontraditional symptoms as a group, and mental status change, in particular, by narrator type and sex.

## Results

We obtained data from 191 stroke stories that met the inclusion criteria, with 52.4% (100/191) about female stroke patients and 47.6% (91/191) about male stroke patients. Of these stories, 174 reported symptoms (52.3%, 91/174 for women patients; 47.7%, 83/174 for men patients). Of the 174 stories reporting symptoms, 85 (48.9%) stories included age. Table 1 presents the age distribution by sex. Given the age distribution of bloggers [26], our sample overrepresented younger adults, yielding proportionately more reports about patients aged 45 to 64 years than 65 years and older. This contrasts with the monotonic increase in risk of stroke that occurs with age during adulthood [17,19]. However, 89 stories (51.1%) did not include age and it is unknown if the patients in these stories had an age distribution similar to Table 1.

Of the 191 stories, 50.8% (97/191) were narrated by the patient and 49.2% (94/191) were narrated by a third person. These percentages did not differ by sex of the patient, as shown by chi-square analysis (Table 2). When a third person was the narrator, he or she was a witness to the stroke for 39.6% (19/48) of women and 45.7% (21/46) of men, a nonsignificant sex difference. Most (83%, 78/94) third-person narrators were a relative of the patient and the type of relative varied for men and women patients in the chi-square analysis (*P*=.008). The narrators for women patients were more likely to be their adult child (60.0%, 24/40) or other relative (25.0%, 10/40) than for men patients (39.5%, 15/38 and 13.2%, 5/38, respectively). Men patients were more likely to have their spouse as narrator (47.4%, 18/38) than women patients were (15.0%, 6/40).

**Table 1:** Age distribution for women (n=40) and men (n=45) patients reporting age.

Age (years)	Patients, n (%)	
	Women	Men
0-17	2 (5)	1 (2)
18-29	9 (23)	5 (11)
30-44	16 (40)	8 (18)
45-64	8 (20)	19 (42)
65-75	3 (8)	8 (18)
76-84	0 (0)	3 (7)
85+	2 (5)	1 (2)

**Table 2:** Profiles of narrators for women (n=100) and men (n=91) patients.

Narrator characteristics		Patients, n (%)		*P*
		Women	Men	
**Narrator**				.72
	First-person	52 (52.0)	45 (49.5)	
	Third-person	48 (48.0)	46 (51.5)	
**Third-person witness to stroke**				.55
	Yes	19 (39.6)	21 (45.7)	
	No	29 (60.4)	25 (54.3)	
**Third-person relationship-relative**				.008
	Adult child	24 (60.0)	15 (39.5)	
	Other	10 (25.0)	5 (13.2)	
	Spouse	6 (15.0)	18 (47.4)	
**Third-person relationship-nonrelative**				n/a
	Friend	2 (25.0)	3 (37.5)	
	Stranger	6 (75.0)	5 (62.5)	

Chi-square analyses of the number of men and women experiencing specific stroke symptoms showed no significant sex differences for traditional or nontraditional symptoms, as seen in Table 3. The largest sex differences were for mental status change and visual disturbances, with women reporting more of these symptoms, although neither difference reached statistical significance. In a further analysis, we evaluated sex differences in mental status change separately for first- and third-person narrators (the small number of reports of visual disturbances precluded a similar analysis of this symptom). The narrators for the 91 women and 83 men patients included in the symptom analysis were 53% (92/174) first person and 47% (82/174) third person across sex. First-person narrators reported more mental status change than third-person narrators (*P*=.01). When mental status change was analyzed by patient sex (see Table 4), first-person narrators were more likely to report mental status change when the narrator was a woman patient (56%, 27/48) than a man patient (36%, 16/44), although this effect narrowly missed statistical significance (*P*=.056). However, third-person narrators showed no difference in their reports of mental status change for women (28%, 12/43) and men patients (28%, 11/39).

**Table 3:** Stroke symptoms reported for women (n=91) and men (n=83) patients.

Symptom type			Patients, n (%)		*P*
			Women	Men	
**Traditional symptoms**					
	**Hemiparesis/hemiplegia**		68 (74.7)	63 (75.9)	.86
		Body	30 (44.1)	33 (52.4)	.33
		Face	2 (2.9)	4 (6.3)	
		Both body and face	36 (52.9)	26 (41.3)	
	Impaired speech or comprehension		59 (64.8)	47 (56.6)	.27
	Visual disturbance		11 (12.1)	5 (6.0)	.17
	Ataxia, discoordination		16 (17.6)	11 (13.3)	.43
	Vertigo		13 (14.3)	13 (15.7)	.80
	Difficulty with balance		7 (7.7)	8 (9.6)	.65
**Nontraditional symptoms**					
	Pain (excluding headache)		5 (5.5)	5 (6.0)	.88
	Mental status change		39 (42.9)	27 (32.5)	.16
	Headache		16 (17.6)	11 (13.3)	.62
	Lightheadedness		5 (5.5)	3 (3.6)	.55
	Other neurologic symptoms		13 (14.3)	9 (10.8)	.67
	Nonneurologic symptoms		4 (4.4)	5 (6.0)	.63

**Table 4:** Reports of mental status change for women (n=91) and men (n=83) patients by narrator.

Mental status change		Patients, n (%)		*P* value
		Women	Men	
**First-person narrator**				.056
	Yes	27 (56.3)	16 (36.4)	
	No	21 (43.7)	28 (63.6)	
**Third-person narrator**				.97
	Yes	12 (27.9)	11 (28.2)	
	No	31 (72.1)	28 (71.8)	

We tested whether this same pattern of effects for patient sex and type of narrator was found for all other nontraditional symptoms combined, excluding mental status change. With 174 male and female patients combined, more of the 92 first-person narrators (44.6%, 41/92) reported at least 1 nontraditional symptom than the 82 third-person narrators (25.6%, 21/82, *P*=.006). As shown in Table 5, this same pattern was seen for each sex separately with no significant differences between male and female patients in the narrator effect. This pattern of more nontraditional symptoms reported by first- versus third-person narrators was not because third-person narrators reported fewer symptoms in general: 90% or more of both first- and third-person narrators reported traditional symptoms for both men and women patients.

**Table 5:** Reports of nontraditional symptoms (excluding mental status change) for women (n=91) and men (n=82) patients by narrator.

Number of nontraditional symptoms		Patients, n (%)		*P*
		Women	Men	
**First-person narrator**				.80
	≥1	22 (45.8)	19 (43.2)	
	None	26 (54.2)	25 (56.8)	
**Third-person narrator**				.62
	≥1	12 (27.9)	9 (23.1)	
	None	31 (72.1)	30 (76.9)	

Of the stories describing symptoms, 96 reported a medical diagnosis of an ischemic (n=67) or hemorrhagic (n=29) stroke. We compared the number of reports of each traditional and nontraditional symptom by ischemic and hemorrhagic stroke patients (see Table 6). Chi-square analysis showed differences between the 2 stroke types for 2 specific symptoms: ischemic stroke patients were significantly more likely to experience vertigo (*P*=.008), whereas hemorrhagic stroke victims were significantly more likely to experience headaches (*P*=.002). Categorizing stroke symptoms as traditional or nontraditional symptoms, 79% (23/29) of hemorrhagic stroke patients experienced nontraditional symptoms whereas only 54% (36/67) of ischemic stroke patients did (*P*=.001). Looking at percentage of patients in Table 6, symptoms of general pain, mental status change, and headache seemed to contribute most to this effect of stroke type on nontraditional symptoms.

**Table 6:** Reports of stroke symptoms by ischemic (n=67) and hemorrhagic (n=29) stroke.

Symptom type			Stroke type, n (%)		*P*
			Ischemic	Hemorrhage	
**Traditional symptoms**					
	**Hemiparesis/hemiplegia**		56 (84)	20 (69)	.11
		Body	19 (34)	9 (45)	.30
		Face	5 (9)	0 (0)	
		Both body and face	32 (57)	11 (55)	
	Impaired speech or comprehension		42 (63)	19 (66)	.79
	Visual disturbance		8 (12)	3 (10)	.82
	Ataxia, discoordination		13 (19)	2 (7)	.12
	Vertigo		18 (27)	1 (3)	.01
	Difficulty with balance		5 (7)	2 (7)	.92
**Nontraditional symptoms**					
	Pain (excluding headache)		3 (4)	4 (14)	.11
	Mental status change		21 (31)	14 (48)	.11
	Headache		7 (10)	11 (38)	.002
	Lightheadedness		5 (7)	0 (0)	.13
	Other neurologic symptoms		10 (15)	5 (17)	.77
	Nonneurologic symptoms		5 (7)	2 (7)	.92

Virtually all stroke patients sought medical assistance (96%-97%) regardless of sex, although 44% (35/80) of women patients delayed getting assistance and 32% (24/76) of men patients delayed, a difference that did not reach statistical significance (*P*=.12). There was no sex difference in time to get treatment in the hospital; 90% to 91% of both women and men patients received treatment immediately upon arrival.

## Discussion

### Principal Findings

The results demonstrate that weblogs are a useful source of stories of stroke experiences and that most stories describe symptoms. The reliability of the description of experiences in the weblog stories is supported by their replication of several previous findings in studies based on hospital interview data: the more frequent reports of traditional than nontraditional symptoms [15], evidence that women patients experience mental status change (here in the analysis by narrator) as a symptom more than men [11,13,15,18,22,23], the greater frequency of ischemic than hemorrhagic strokes [8,11,15], and the greater frequency of headaches and other nontraditional symptoms in hemorrhagic strokes [25,32].

The results also offer some new findings involving variables that affect symptom reports and sex differences, but that have been relatively unexplored in previous studies. As hypothesized, mental status change was reported more by first- than third-person narrators. The most obvious explanation is that this symptom is a change in an internal state that the patient is aware of but may not be visible to a third-person observer, unlike other symptoms that involve observable effects (eg, hemiplegia, impaired speech). Moreover, the frequency of report of mental status change showed no sex difference with third-person narrators, but was greater for women than men patients with first-person narrators, although this missed statistical significance (*P*=.056). Further research is needed to follow up this finding with a study including a greater number of patients that will allow control of variables confounded with sex in the present study, most importantly age. The age distribution suggests that the sample of women patients was younger than the sample of men patients, although narrators did not consistently supply information on patient age. Nonetheless, the narrator effect is consistent with the hypothesis that mental status change may be more available to the patient than to third-person narrators because it is an internal state.

Another significant effect of narrator was on reports of nontraditional symptoms. First-person narrators produced more nontraditional symptoms than third-person narrators, excluding the symptom of mental status change. This difference may be because other nontraditional symptoms involve internal states and are difficult for a third party to observe (eg, headache, pain). Thus, the results suggest that a first- or third-person narrator significantly affects the type of symptom reported and also whether or not sex differences are observed for symptoms, such as mental status change.

Third-person narrators are frequently called upon to describe symptoms to first responders or hospital personnel. Indeed, first- and third-person narrators are common in studies of stroke symptoms based on hospital interviews or records (eg, [11,15,16,18,23]). The present study, however, is the first to suggest that type of narrator is an influential variable and to demonstrate its significant association with specific symptoms. Previous studies have not evaluated effects of type of narrator. The present results suggest that type of narrator is an important variable to include in analysis of symptoms in hospital stroke studies, especially in the analysis of sex differences.

### Limitations

The results also demonstrate the limitations of using weblogs as a source of data. Important information about the patient, especially age, was often not reported. The stories were from public blogs and written by people who were not given specific instructions about which details to include. This creates a fragmentary data problem that is inherent in this type of research. Additionally, the StoryUpgrade system does not provide a complete picture of all stroke experiences narrated on the Web. The system does not find all stories posted to weblogs, and as new technologies emerge for sharing personal experiences (eg, platforms such as Twitter and Facebook), additional techniques will be required to capture these stories. With respect to patient age, the distribution of ages that were included in stories showed overrepresentation of younger adult patients and very few patients older than 76 years. This contrasts with the monotonic increase in risk of stroke that occurs with age during adulthood [17,19]. The atypical representation of relatively young adult patients in weblogs may reflect, in part, the surprise and shock when a relatively young adult suffers a stroke, an illness associated with old age. Such an emotional response may motivate a blog. The most important factor for the observed age distribution in this study, however, is likely that younger adults use weblogs more than older adults [26] and adults aged 65 years and older use the Internet substantially less than younger adults.

### Conclusions

Our results demonstrate that our method of using weblogs as a source of data on stroke experiences can produce interesting new findings that have important implications for understanding sex differences and that generate hypotheses to test in future research. Older adults’ rapidly increasing use of the Internet [33] suggests that their representation in future Internet research will only grow, reducing a limitation of the present research. Using weblogs to collect data for medical research has the advantage of being relatively fast and inexpensive because it greatly reduces the time and cost of gathering data, relative to hospital studies. The price for this, however, is that data are fragmentary without a standard format for generating data.

Finally, the description of symptoms in blogs is important in terms of testing sex differences, but also for the insight it provides into the nature of stroke symptoms communicated to people who frequent weblogs or search the Internet to obtain information about strokes. That is, the Internet has become a key destination for people seeking information about disease [34]; thus, stories in blogs are likely to influence how people conceptualize stroke symptoms.

Overall, we see an important place for techniques such as these in medical research. They provide health scientists with a useful effective tool to explore medical issues. Very quickly, a health researcher can examine the experiences of people confronted with a medical issue of interest. Although these experiences will be colored by biases introduced by the query process and the population of bloggers, the information gleaned may be useful to formulate new questions and hypotheses that warrant additional investigation. Without the time and expense required for a full-scale traditional hospital study, health professionals can leverage the experiences of Web users to formulate promising avenues for future research.

## Multimedia Appendix 1

Prototype story used for initial search query of blogs.
