# Supplementary material for: Stroke Experiences in Weblogs: A Feasibility Study of Sex Differences
Source: J Med Internet Res. 2014 Mar 19;16(3):e84. doi: 10.2196/jmir.2838 (PMC3978549; doi:10.2196/jmir.2838)
Supplement: Supplementary file 1 [file jmir_v16i3e84_app1.pdf]

Prototype Story Used for Initial Search Query of Blogs

I was watching TV and I slumped over on my side. I just didn’t feel right. I felt weak and I didn’t feel normal on one side. I had a hard time speaking and I could not find the words I wanted to use. My speech did not make sense and was slurred. I felt confused and could not pick up my arm or leg on one side. I felt anxious and upset. I felt scared. My head felt strange. My friend called 911. The paramedics arrived. I was loaded into the ambulance and taken to the emergency room. There, at the triage unit I was diagnosed with a stroke.
